# In situ Detection of Cobaloxime Intermediates During Photocatalysis Using Hollow‐Core Photonic Crystal Fiber Microreactors

**DOI:** 10.1002/anie.202214788

**Published:** 2023-01-18

**Authors:** Takashi Lawson, Alexander S. Gentleman, Jonathan Pinnell, Annika Eisenschmidt, Daniel Antón‐García, Michael H. Frosz, Erwin Reisner, Tijmen G. Euser

**Affiliations:** ^1^ NanoPhotonics Centre Cavendish Laboratory University of Cambridge JJ Thomson Avenue Cambridge CB3 0HE UK; ^2^ Yusuf Hamied Department of Chemistry University of Cambridge Lensfield Road Cambridge CB2 1EW UK; ^3^ Max Planck Institute for the Science of Light Staudtstr. 2 91058 Erlangen Germany

**Keywords:** Cobaloximes, Hollow-Core Photonic Crystal Fibers, Laser Spectroscopy, Optofluidics, Photocatalysis

## Abstract

Hollow‐core photonic crystal fibers (HC‐PCFs) provide a novel approach for in situ UV/Vis spectroscopy with enhanced detection sensitivity. Here, we demonstrate that longer optical path lengths than afforded by conventional cuvette‐based UV/Vis spectroscopy can be used to detect and identify the Co^I^ and Co^II^ states in hydrogen‐evolving cobaloxime catalysts, with spectral identification aided by comparison with DFT‐simulated spectra. Our findings show that there are two types of signals observed for these molecular catalysts; a transient signal and a steady‐state signal, with the former being assigned to the Co^I^ state and the latter being assigned to the Co^II^ state. These observations lend support to a unimolecular pathway, rather than a bimolecular pathway, for hydrogen evolution. This study highlights the utility of fiber‐based microreactors for understanding these and a much wider range of homogeneous photocatalytic systems in the future.

## Graphical abstract

Catalytic intermediates can be observed on microliter volumes using in situ fiber‐based spectroscopy. The hydrogen‐evolving reaction driven by the combination of cobaloxime hydrogen evolution catalysts with [Ru (bpy)_3_]^2+^ photosensitizers reveal the formation of a transient Co^I^ species and a steady‐state Co^II^ species.

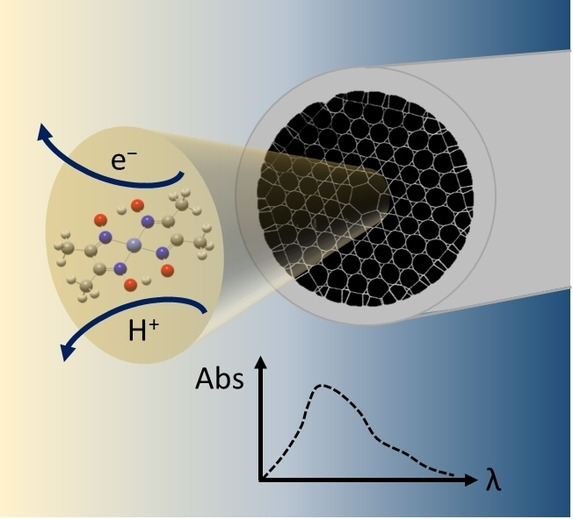

## Introduction

The development of artificial photosynthesis, inspired by natural photosynthesis, has evolved over the past few decades as a promising approach to harvest and store solar energy in renewable fuels and chemicals. Model artificial photosynthetic systems generally involve a light‐harvester (i.e., a photosensitizer) and a co‐catalyst in solution or suspension with a sacrificial electron donor (SED), and acceptor substrates (e.g., CO_2_, H^+^, H_2_O). Studying the interplay between all these components allows optimization and enhancement of efficiency, with many investigations aiming at understanding particular aspects of the system, e.g., the electron transfer dynamics between the photosensitizer and co‐catalyst. To this end, routine quantitative analyses of photocatalysts have included electrochemical measurements,[^1^, ^2^] fluorescence quenching (Stern–Volmer) analysis,[^1^, ^3^] transient absorption spectroscopy,[^4^, ^5^] and measurement of product yields.^[6]^ However, these methods give little mechanistic insight into the sequence of steps that enable the full photocatalytic reaction to proceed.

Mechanistic studies of multistep photocatalytic reactions often relate to elucidating rate‐limiting steps in a catalytic cycle.[^6^, ^7^] These investigations focus on characterizing catalytic intermediates either by isolation,[^8^, ^9^] spectroscopic identification,[^1^, ^10^, ^11^] or through computational methods based for example on density functional theory (DFT).[^12^, ^13^] Experimentally identifying catalytic intermediates remains challenging as these species typically exist on limited time scales and at very low concentrations.[^14^, ^15^, ^16^, ^17^] Therefore, such studies strongly benefit from the long optical path lengths and thus enhanced sensitivity offered by microreactors based on a hollow‐core photonic crystal fiber (HC‐PCF); a special type of optical fiber that uses interference effects to guide light along a microfluidic channel.^[18]^

**Figure 1:**
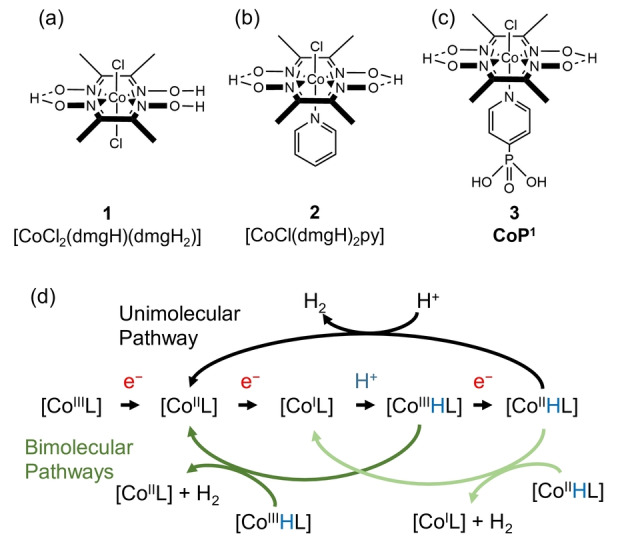
Cobaloxime catalysts for hydrogen evolution. a) [CoCl_2_(dmgH)(dmgH_2_)] (**1**), b) [CoCl(dmgH)_2_py] (**2**), and c) **CoP^1^** (**3**). d) The unimolecular reaction mechanism for hydrogen generation via proton reduction by cobaloxime catalysts.[^29^, ^31^, ^32^, ^48^, ^49^] L represents the ligands. Bottom: Alternative bimolecular pathways comprise two Co^III^‐hydrides interacting to form two Co^II^ species and H_2_, or two Co^II^‐hydrides interacting to form two Co^I^ species and H_2_.[^29^, ^32^] Charges omitted for clarity.

Many artificial photosynthetic systems comprising a photosensitizer in conjunction with a hydrogen evolution co‐catalyst have been proposed for H_2_ generation from water.[^19^, ^20^, ^21^] One such system consists of a [Ru(bpy)_3_]^2+^ (bpy=2,2′‐bipyridine) light absorber and a cobaloxime co‐catalyst,^[20]^ which is an inexpensive alternative to platinum, hydrogenases and other molecular catalysts for the reduction of protons.^[22]^ Cobaloximes are cobalt‐based metal complexes containing equatorial dimethylglyoximato (dmgH^−^) ligands (**1‐3**, Figure 1a–c), shown to be effective proton reduction catalysts in aqueous solutions.[^23^, ^24^] Molecular catalysts based on 3*d* transition metals (i.e., cobalt, nickel, iron) have the potential to be produced at low cost and scale, display tunability through ligand design and modification, and possess high atom efficiencies with full utilization of metal centers for catalysis.[^19^, ^25^, ^26^] For example, the simple addition of an axial pyridine ligand (**2**, Figure 1b) and tuning of electronics by derivatization enhances the activity of cobaloxime catalysts.[^23^, ^27^] Moreover, the addition of a phosphate group on the pyridine axial ligand to form **CoP^1^** (**3**, Figure 1c) enables good solubility in water.[^27^, ^28^]

Initially, in the generally accepted mechanism for H_2_ evolution using cobaloxime catalysts, two one‐electron reduction steps of Co^III^ give the Co^I^ state, which is followed by protonation to produce a metal‐hydride, Co^III^‐H that is widely regarded as the rate‐limiting step.[^29^, ^30^, ^31^] From here, there are different catalytic pathways proposed for H_2_ formation. Experimental and computational studies have suggested that an additional electron transfer to Co^III^‐H could form a Co^II^‐H species, which is then protonated a second time to release H_2_ and Co^II^ via a monomolecular pathway. Alternatively, a bimolecular pathway with two Co^III^‐H species or two Co^II^‐H species could produce H_2_.^[32]^ The proposed electron and proton transfer steps are summarized in Figure 1d, but conclusive evidence of these mechanisms remains difficult to collect as they generally rely on experimental conditions and techniques that can only analyze the reaction once it has reached a steady‐state equilibrium or completion (e.g., UV/Vis spectroscopy). Some techniques can analyze the reaction dynamics on femtosecond to nanosecond timescales (e.g., transient absorption spectroscopy), which can lead to some mechanistic insights. However, the excitation pulses are much shorter and have light intensities that are much higher than those used under normal operating conditions. Thus, such methods do not always reveal all the important reaction intermediates that exist under normal operation. Sometimes spectroscopic investigation requires high concentrations, and this may alter the actual mechanism. In this case, a higher concentration will start favouring a bimolecular mechanism.

In situ methods under actual reaction conditions are required to identify the nature of catalytic intermediates, and therefore provide utility in elucidating the correct reaction mechanism. Importantly, cobaloximes have characteristic absorption spectra dependent on the oxidation state of the cobalt metal center.[^32^, ^33^, ^34^] Therefore, in situ spectroscopy can be used to identify these intermediate states with the enhanced sensitivity of fiber‐based microreactors on sample volumes smaller than one microliter. High‐sensitivity UV/Vis absorption spectroscopy in HC‐PCF microreactors has already found applications in a wide range of chemical sensing applications.[^35^, ^36^, ^37^] More recently, HC‐PCF microreactors have been combined with fluorescence methods to detect singlet‐oxygen with sub‐picomole sensitivity,^[38]^ and to perform Stern–Volmer analysis on sub‐μL catalyst volumes.^[39]^ Finally, Raman spectroscopy has been used to monitor reaction mixtures[^40^, ^41^] and liquid electrolytes^[42]^ in situ.

The Co^I^ state, stabilized by the cobaloxime ligands, has been detected in a dichloromethane (DCM) solvent system for the selective synthesis of alkenylphosphine oxides under visible light,^[43]^ and also by spectroelectrochemical methods.[^30^, ^34^, ^44^] The Co^I^ state has been detected at pH 12 in aqueous solvents,^[45]^ but it has not been detected in situ at appropriate pH for hydrogen evolution. This is due to the low absorption at relevant concentrations and as such, cannot be measured easily with conventional techniques.

Here we use recently developed microreactor technology based on HC‐PCFs for the in situ monitoring of various oxidation states of cobaloximes accessed during proton reduction and demonstrate the utility in supporting a catalytic mechanistic pathway.

## Results and Discussion

**Figure 2:**
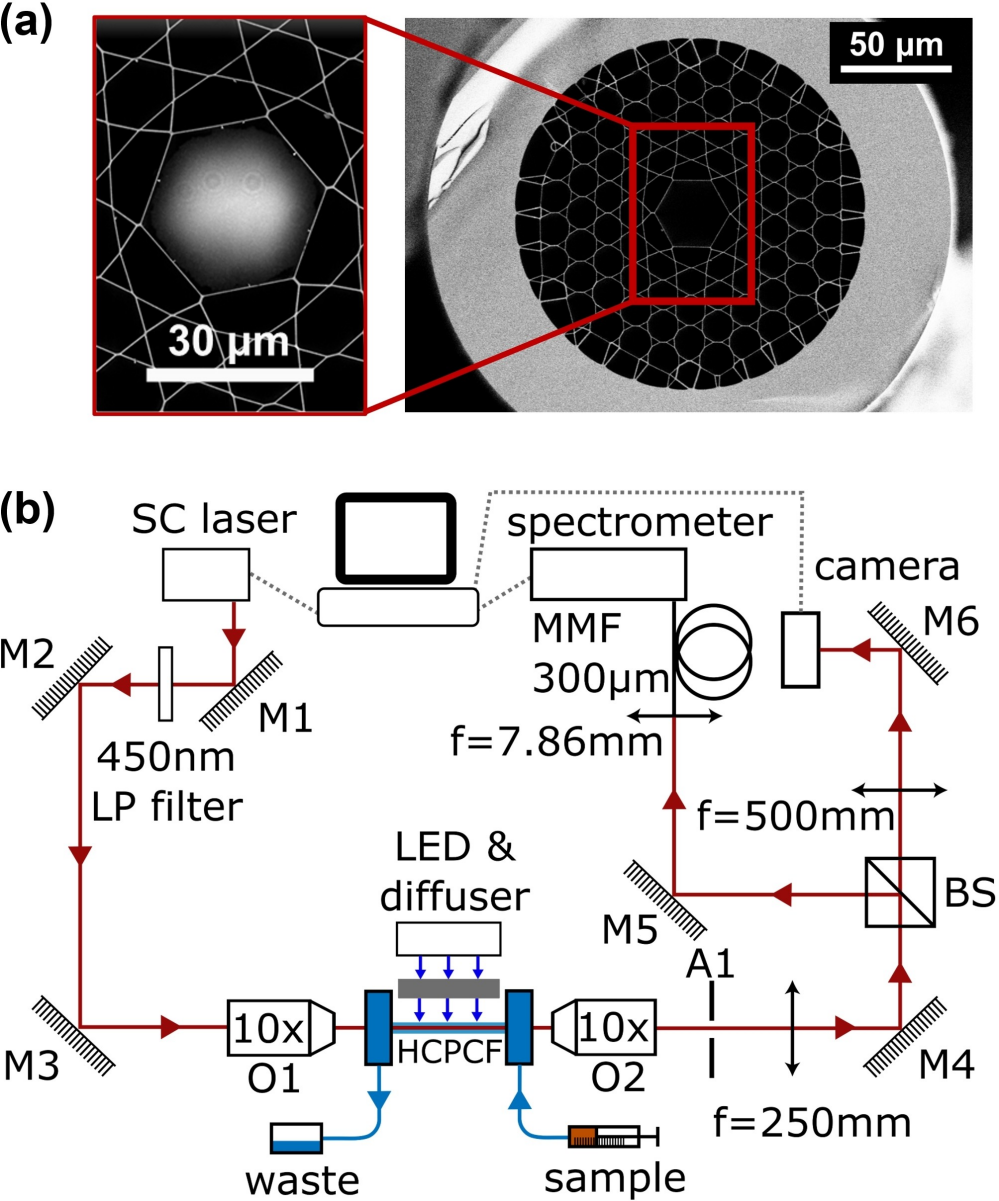
a) Scanning electron microscopy (SEM) image of the core region of the HC‐PCF overlaid with the fundamental LP_01_ optical mode intensity profile utilized for electronic absorption spectroscopy. b) Optical setup for in situ spectroscopy. BS, beamsplitter; LP, long‐pass; MMF, multimode fiber (300 μm core diameter); SC, supercontinuum. O1 corresponds to the in‐coupling objective lens and O2 corresponds to the out‐coupling objective lens. A1 is an aperture used for alignment.

Cobaloximes were synthesized and characterized via protocols outlined in previous work,[^28^, ^46^] and [Ru(bpy)_3_]Cl_2_ was purchased from a commercial supplier. These were spectroscopically characterized within kagomé‐style HC‐PCFs with two excitation sources (*λ*_ex_=365 nm, 450 nm, see Supporting Information, Figure S1) in the same manner as reported in previous work.^[37]^ Kagomé‐style HC‐PCFs (Figure 2a), a PCF type first used by Benabid et al.,^[47]^ were employed as their guidance mechanism permits broadband transmission extending from the ultraviolet (UV) to the near‐infrared in water and hence, are the preferred choice of HC‐PCF for broadband UV/Vis spectral measurements.^[36]^ The kagomé‐style HC‐PCF was drawn to a core diameter and wall thickness of 30 μm and 160 nm, respectively, resulting in a reaction volume of less than 10 nL per cm of fiber. The 30 μm core diameter allows for good optical coupling efficiency, with the surrounding kagomé lattice providing excellent mechanical stability. Mechanical stability minimises bending‐induced transmission losses of the probe light. A broadband supercontinuum laser source (NKT SuperK Compact) was coupled into the HC‐PCF, exciting a fundamental LP_01_ mode, which was used to obtain an electronic absorption spectrum. Excitation of the photosensitizer (*λ*_max_=450 nm) by the probe source was limited by employing a 450 nm long pass filter and reducing the incident power to below 3 μW. The optical setup is shown in Figure 2b. Samples were purged and infiltrated into the fiber under a nitrogen atmosphere. Optimum conditions to observe a photoinduced absorption signal were identified, and as such, three different cobaloximes were characterized under the same reaction conditions to identify subtle differences in the absorption spectra and reaction kinetics.

### Comparing Reaction Conditions

**Figure 3:**
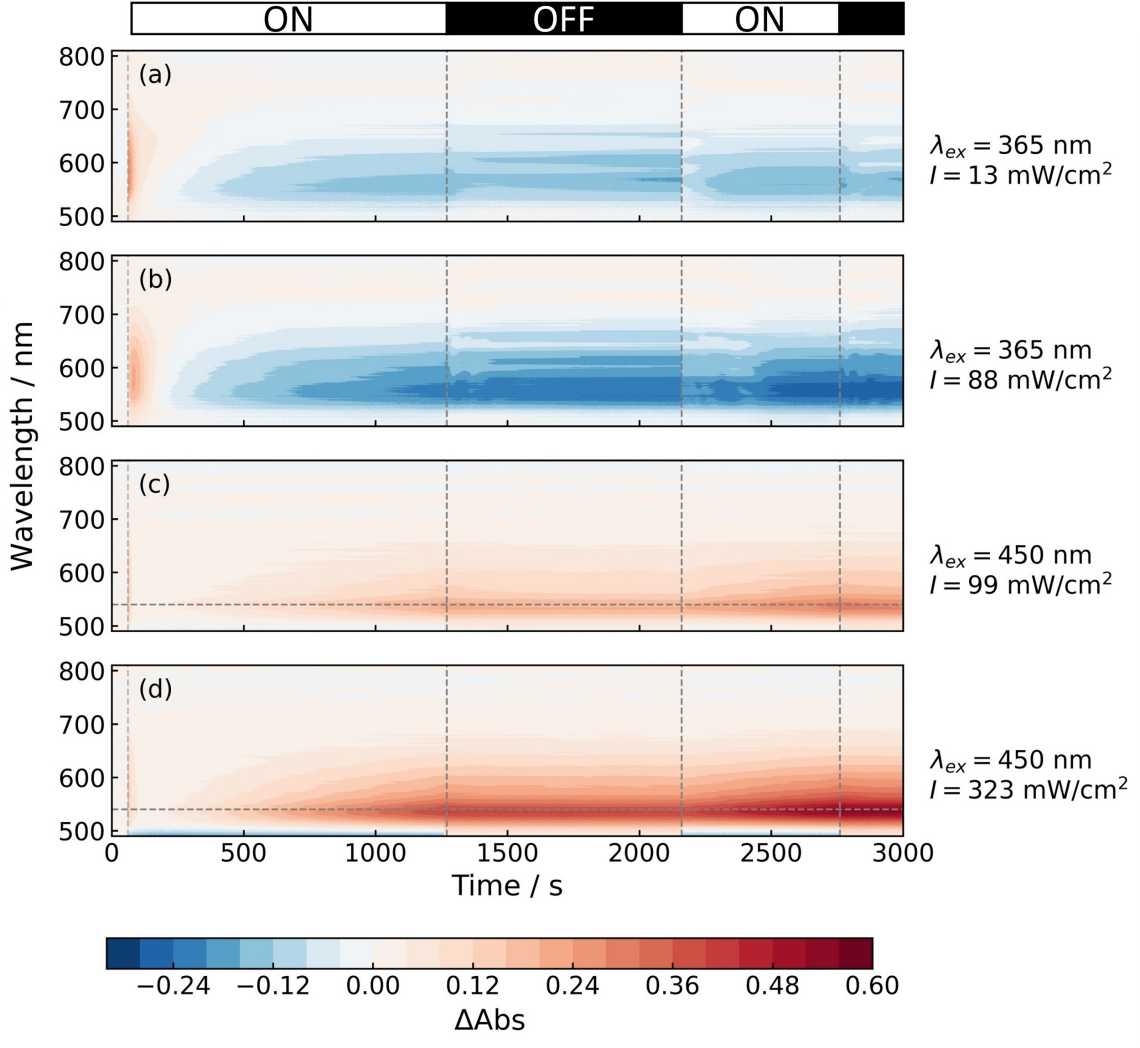
*In‐fibra* color maps detailing the time evolution of absorption as a function of wavelength for **3** with [Ru(bpy)_3_]^2+^ as a photosensitizer and sodium ascorbate as an electron donor. Each sample was made up of 44 μM [Ru(bpy)_3_]^2+^, 86 μM cobaloxime **3**, 0.1 M sodium ascorbate and held at pH 8 using a phosphate buffer (0.2 M). Profiles (a, b) were driven by a 365 nm LED source and (c, d) a 450 nm LED source. The absorption traces at 540 nm, indicated by the horizontal lines in (c, d), are plotted in Figure 4e. The horizontal bar at the top of the figure shows the times at which the LED source was switched on or off.

Samples containing [Ru(bpy)_3_]Cl_2_ (44 μM), cobaloxime **3** (86 μM) with sodium ascorbate (0.1 M) as SED[^20^, ^50^, ^51^] in pH 8 phosphate buffer (0.2 M) were prepared, with the results shown in Figure 3. The [Ru(bpy)_3_]^2+^ triplet excited state undergoes reductive quenching by the ascorbate ion, with subsequent electron transfer from the photogenerated [Ru(bpy)_3_]^+^ species to the cobaloxime catalyst.^[20]^ Although the optimum pH for H_2_ evolution for this system is pH 5,^[20]^ cobaloximes have been reported to work best between pH 7 and pH 12 for other systems.[^29^, ^45^, ^52^] The mismatch in optimal pH is the result of the different solvents and SEDs employed. For all our experiments, alkaline conditions were employed to limit H_2_ turnover and build up the population of catalytic intermediates.

A rapid photo‐induced absorption signal (PIA) is observed upon excitation (Figure 3a–d). The PIA disappears within one minute. This is indicative of an electron transfer event, and the formation of a new species. This species is postulated to be the Co^I^ intermediate, which most likely arises due to the reductive quenching of an excited state of [Ru(bpy)_3_]^2+^. Importantly, the small magnitude of this absorption (0.1) was only detectable over a 5 cm path length, allowing us to use dilute, realistic conditions. A conventional 1 cm cuvette measurement yielded the PIA with much lower signal‐to‐noise (abs.<0.03) for cobaloximes in general (Supporting Information, Figure S2).

Following the disappearance of the PIA, the depletion of a ground state species absorbing at a *λ*_max_=560 nm is apparent when the photoexcitation is driven at 365 nm (Figure 3a,b), whereas the emergence of a new absorbing species with a *λ*_max_=540 nm is observed when the photoexcitation is driven at 450 nm (Figures 3c,d). The ground state bleach at *λ*_ex_=365 nm can be attributed to **3** as this behaviour is not observed when methyl viologen is employed as the electron acceptor.^[37]^ Moreover, the spectral shape of the bleach matches the absorption tail of the Co^III^ oxidation state of **3** (Supporting Information, Figure S3a), and ascorbate degradation products are not expected to absorb in the visible region. Degradation of catalyst **3** via reactive oxygen species becomes more likely with UV excitation. The magnitude of the absorbance does not change when the UV source is switched off, confirming that the degradation is UV‐driven.

The population of the new absorbing Co species with a *λ*_max_=540 nm (Figure 3c, d, *λ*_ex_=450 nm) rises linearly with time when the 450 nm LED source is on and remains stable at a fixed absorbance when the 450 nm LED source is switched off. This suggests that this absorbing species is generated by a light‐driven process and is stable in a nitrogen‐purged solution. As such, optimal conditions for monitoring the various oxidation states of cobaloximes involve using a 450 nm LED source to initiate the reduction of the cobalt(II/III) center and to avoid degradation of the catalyst. More broadly, these measurements highlight the utility of HC‐PCFs in screening for the best reaction conditions.

### Comparing Cobaloximes

**Figure 4:**
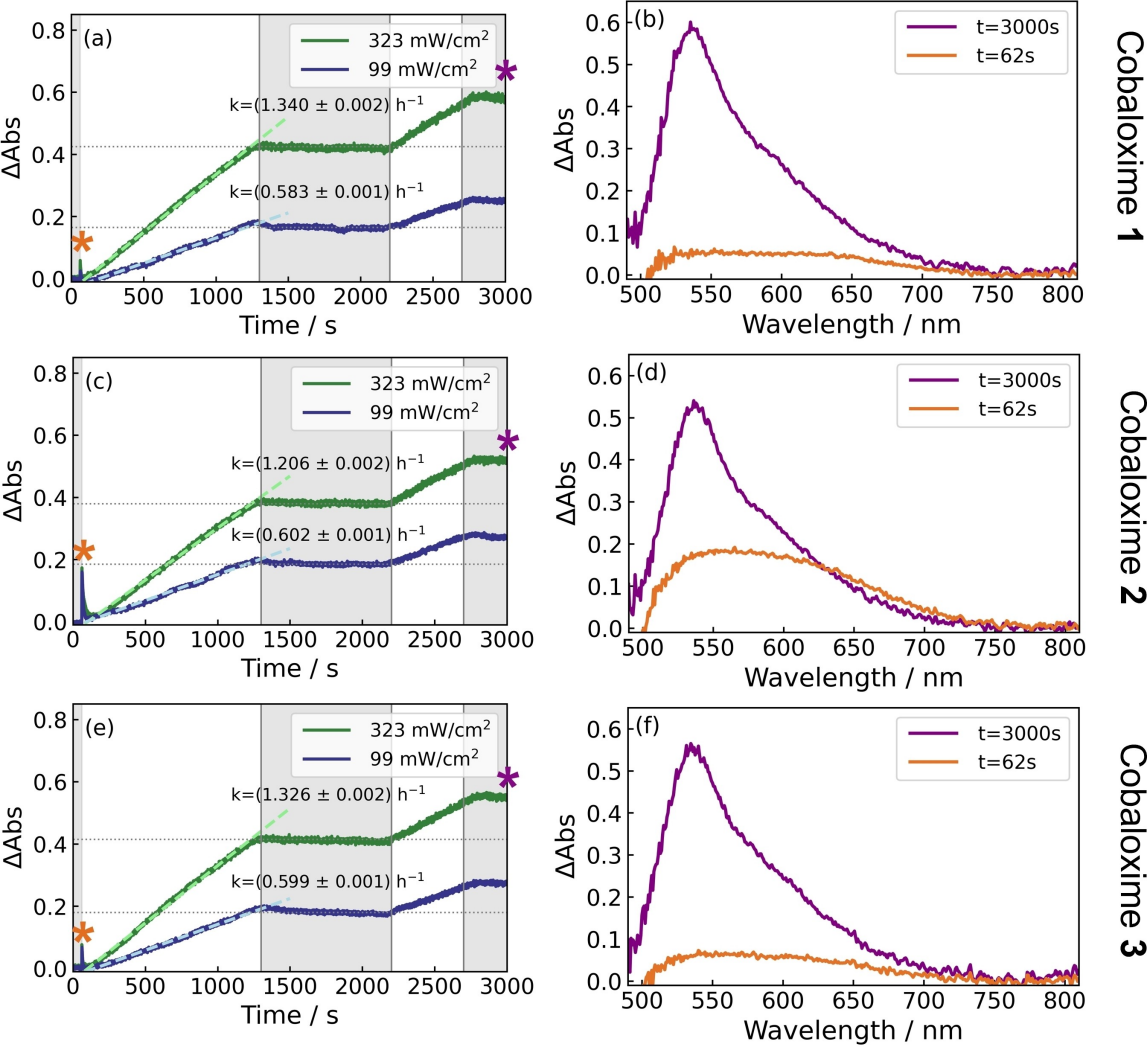
Photoinduced absorption profiles of three cobaloximes with axial chlorido ligands (**1**, a, b), an axial pyridine ligand (**2**, c, d) and an axial phosphonate ligand (**3**, e, f) under *λ*_ex_=450 nm irradiation. Absorption time traces (a, c, e) are taken at 540 nm under 99 mW cm^−2^ and 323 mW cm^−2^ irradiances, with a linear fit applied to extract a zero‐order rate constant. The excitation source was switched on at *t*=60 s, off at *t*=1260 s, on at *t*=2160 s and off at *t*=2760 s. Irradiated and non‐irradiated times are indicated by the white and gray background shading, respectively. Spectral profiles (b, d, f) are shown for each cobaloxime studied at *t*=62 s (indicated by the orange asterisks in a, c, e) and *t*=3000 s (indicated by the purple asterisks in a, c, e) under an irradiance of 323 mW cm^−2^. These correspond to the initial photoinduced absorption of a Co^I^ species, and the growing population of a Co^II^ species, respectively. The absorption traces in (e) correspond to the spectral maps shown in Figures 3c and d.

Three different cobaloximes were next compared at a concentration of 86 μM with sodium ascorbate (0.1 M) and [Ru(bpy)_3_]Cl_2_ (44 μM) in pH 8 phosphate buffer (0.2 M). Samples were photoexcited with a 450 nm source (99 mW cm^−2^ or 323 mW cm^−2^). The absorption time traces at 540 nm, together with the spectral profiles at key times, are shown in Figure 4. The three different cobaloximes consisted of **1**, **2** where the latter was self‐assembled by adding 1 equivalent of pyridine to cobaloxime **1**,^[23]^ and **3** with a phosphate anchor on the pyridine axial ligand.

**Figure 5:**
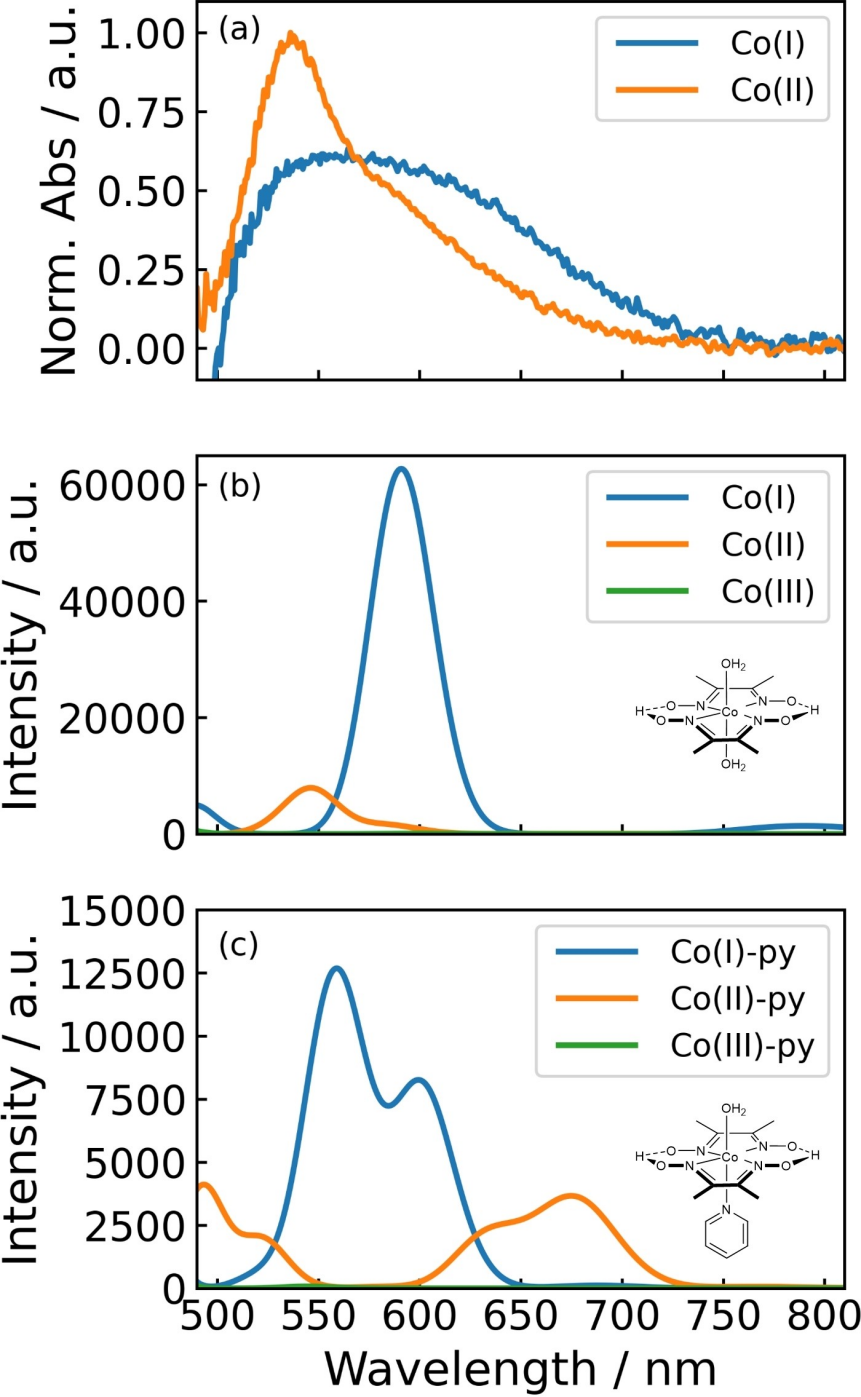
Comparison of DFT‐simulated spectra to spectra obtained *in‐fibra*. a) Normalized experimental spectra of the Co^I^ transient species and Co^II^ steady state species for the [CoCl(dmgH)_2_py] catalyst. b) DFT‐simulated spectra for a cobaloxime with two axial aqua ligands. c) DFT‐simulated spectra for a cobaloxime with one aqua ligand and one pyridine ligand. Charges omitted for clarity.

As discussed above, the time‐resolved absorbance for each cobaloxime consists of a transient component (Figure 4b,d,f–*t*=62 s) and a steady‐state component that increases linearly with irradiation time (Figure 4b,d,f–*t*=3000 s). The transient component (*t*=62 s) absorbs broadly in the range 500 nm to 700 nm. It is most prominent for the cobaloxime with the pyridine group attached (see Figure 4d). The steady‐state component (*t*=3000 s) has a narrower absorbance profile, with a *λ*_max_=540 nm and a shoulder peak evident at 570 nm. The steady‐state component grows at a similar rate with all three Co species under 99 mW cm^−2^ irradiation. However, a lower rate is observed with **2** compared to the other Co catalysts under 99 mW cm^−2^ irradiation. DFT simulations using the B3LYP functional and 6‐31G(d,p) basis set combination were performed to assign chemical species to experimentally measured UV/Vis spectra (Figure 5a).

DFT‐simulated UV/Vis spectra are presented in Figures 5b–c (methods are described in the Supporting Information). The native Co^III^ state of **3** has two axial substituents (Cl^−^ and the pyridine group with phosphonate anchor), with a reduction to lower oxidation states leading to Cl^−^ dissociation and leaving a free site for catalysis and the formation of a stable Co^II^ state.^[23]^ When the cobaloximes are dissolved and reduced in aqueous solutions (as done so here), labile Cl^−^ is displaced by aqua ligands that offer back‐bonding, based on the significantly higher concentration of water as compared to Cl^−^ in the buffer. As such, DFT simulations were run with aqua ligands replacing chlorido ligands.

DFT simulations predict limited absorption in the visible region for the Co^III^ species. The Co^III^ state relates to the ground state of cobaloximes, whose absorbance was experimentally measured (Supporting Information, Figure S3a). The oscillator strength from the DFT simulated spectra can be carefully interpreted as a relative measure for comparing the absorption coefficients.

Absorption from Co^III^ states is expected to be ×8 weaker than the Co^II^ state and ×60 weaker than the Co^I^ state. The Co^II^ state without a pyridine ligand (i.e., completely labilized) is predicted to have a peak absorbance at 550 nm with a shoulder at 570 nm. This spectral shape matches well with the steady‐state component of absorption observed in experiments, and therefore the absorption observed can be assigned to the Co^II^ oxidation state. DFT simulations of hydride species did not match well with experimental spectra (Supporting Information, Figure S5).

Although the addition of a pendant pyridine ligand is predicted to perturb the absorption profile of the Co^II^ species (Figure 5c), this was not observed in the experiments. This could be due to a combination of factors, such as the incomplete self‐assembly of pyridine ligands with Co metal complexes in solution to start with, and/or poor binding efficiency of the pyridine to the Co^II^ metal center, resulting in the pyridine group detaching and re‐attaching to the Co metal center as part of the photocatalytic cycle.^[23]^ Comparing the spectra from DFT (Figure 5) and the experiment provides strong support for the steady‐state formation of Co^II^ without an axial pyridine ligand, with irradiation time.

The Co^I^ state is quadratically planar in its lowest energy configuration and expected to absorb the most strongly out of the three oxidation states considered. An absorbance centered at 580 nm is expected with a ×6 stronger signal than the Co^II^ state (Figure 5b). The addition of a pyridine group in cobaloxime **2** results in a sharper absorption peak for the Co^I^ state (Figure 4d). This can be explained by a sub‐population of Co^I^ species with a pyridine group attached. Therefore, the pyridine likely detaches from the Co^II^ metal center and re‐attaches to the Co^I^ metal center during the photocatalytic cycle.

The transient signal is most likely the Co^I^ state that disappears within a minute. This assignment is corroborated by the transient signal not being observed at pH 6 (Supporting Information, Figure S4), most likely due to the higher H^+^ concentration leading to more rapid protonation of Co^I^ and thus, a less appreciable population build‐up to be spectroscopically detected.

This is consistent with the observation of the initial rate of reaction of H_2_ production being higher at pH 6 than at pH 8 when a three‐component photocatalytic system comprising a Ru^II^‐based photosensitizer and a cobaloxime hydrogen evolution catalyst is used.^[20]^ In addition, the shape of the absorption spectra assigned to a Co^I^ species by Lazarides et al. and Muresan et al. match well with the broad absorption profiles observed here.[^34^, ^53^] However, in both of these previous studies, a different solvent was used (DCM and DMF, respectively), which could perturb the cobaloxime absorption spectra measured in water.

The absorbance of Co^I^, and hence its population, is greatest with the addition of pyridine (Figure 4d), which lends support to the greater hydrogen turnover observed by Willkomm et al. with this ligand.[^23^, ^27^] The addition of a pendant pyridine, therefore, increases the rate of formation of the Co^I^ state from the Co^II^ state. The Co^II^ state is negligible at early times, which can be explained by its lower oscillator strength and its small population at early times. The absorption from the Co^I^ state likely engulfs the weak Co^II^ signal. Panagiotopoulos et al. report that cobaloximes without pyridine ligands (i.e., with two chlorido ligands) display significantly lower stability than cobaloximes with a pyridine group.^[54]^

Moreover, pyridine ligands with electron‐withdrawing groups (such as a phosphate group) slow down the rate of photocatalytic hydrogen evolution when sensitized by porphyrins.^[23]^ However, the addition of TiO_2_ nanoparticles allowed the attachment of the cobaloxime catalyst to the surface of the TiO_2_ and the catalytic performance was shown to be improved by ×5.^[54]^ The greater population of the Co^I^ state for **2**, evidenced by this species displaying the highest amount of absorption for its transient absorption feature (Figure 4d) relative to its other two cobaloxime counterparts (Figure 4b,f), corroborates the conclusions reported by Panagiotopoulos et al..

## Conclusion

**Figure 6:**
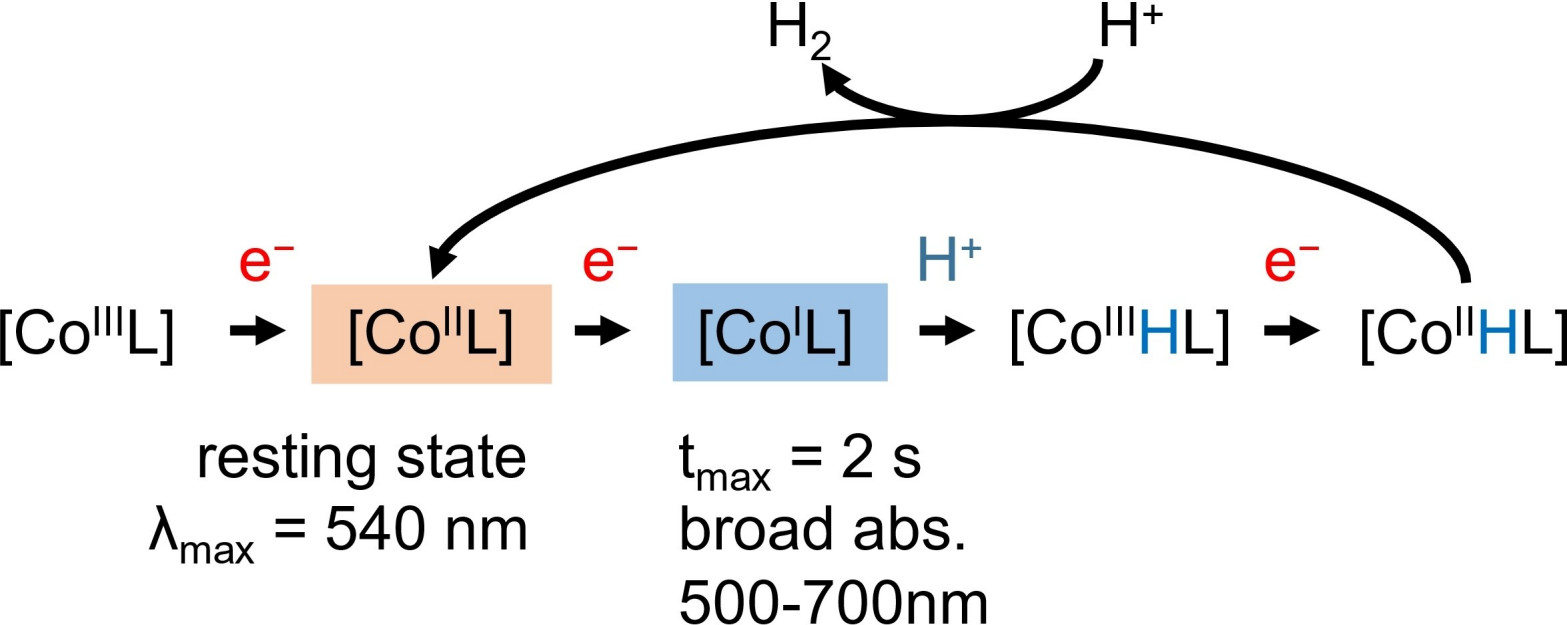
The final proposed reaction mechanism, highlighting the spectral observation of the Co^I^ transient state (blue) and Co^II^ resting state (orange).

We demonstrate that homogeneously dissolved, weakly‐absorbing catalytic intermediates can be observed via novel fiber‐based spectroscopic methods on sub‐microliter volumes in situ. As a model reaction, we chose photocatalytic proton reduction driven by the combination of cobaloxime hydrogen evolution catalysts with a Ru^II^‐based photosensitizer to reveal the formation of a transient Co^I^ species and a steady‐state Co^II^ species. Our studies represent the direct observation of Co^I^ and Co^II^ intermediate species at appropriate pH for hydrogen evolution. Our findings discount the bimolecular pathway forming [Co^I^L] and H_2_ (Figure 1) under the employed experimental conditions, as [Co^I^L] is not the resting state. Furthermore, the low concentration of [Co^III^HL], and the necessity for orientational alignment between two [Co^III^HL], together with the larger thermodynamic requirement for the simultaneous dissociation of two Co^III^−H bonds for H_2_ to evolve, preclude the bimolecular pathway forming [Co^II^L] and H_2_. This lends support to hydrogen evolution via a unimolecular pathway for this photocatalytic system (Figure 6). The addition of an axial pendant pyridine unit was found to increase the stability and absorption of the transient feature assigned to the Co^I^ state.

This proof‐of‐principle study forms a promising basis for future work, for example, using dry organic solvents to artificially accumulate intermediate species. Moreover, this fiber‐based technique could provide further insight into complex multi‐electron molecular catalyzers, such as those employed in CO_2_ reduction, N_2_ fixation or organic photoredox catalysis. The only requirement is that the spectroscopic signature of intermediate species changes with the oxidation state of the metal center. This technique allows spectroscopic tracing under realistic dilute reaction conditions that are often not useable with cuvette‐based analysis.

## Conflict of interest

The authors declare no conflict of interest.

1

## Supporting information

As a service to our authors and readers, this journal provides supporting information supplied by the authors. Such materials are peer reviewed and may be re‐organized for online delivery, but are not copy‐edited or typeset. Technical support issues arising from supporting information (other than missing files) should be addressed to the authors.

Supporting Information

## Data Availability

The data that support the findings of this study are openly available at the Cambridge Data repository.^[55]^
