# Supplementary material for: In situ Detection of Cobaloxime Intermediates During Photocatalysis Using Hollow‐Core Photonic Crystal Fiber Microreactors
Source: Angew Chem Int Ed Engl. 2023 Jan 18;62(9):e202214788. doi: 10.1002/anie.202214788 (PMC10946874; doi:10.1002/anie.202214788)
Supplement: Supplementary file 1 — Supporting Information [file ANIE-62-0-s001.pdf]

Angewandte Chemie
Eine Zeitschrift der Gesellschaft Deutscher Chemiker

Supporting Information

In situ Detection of Cobaloxime Intermediates During Photocatalysis Using Hollow-Core Photonic Crystal Fiber Microreactors

T. Lawson, A. S. Gentleman, J. Pinnell, A. Eisenschmidt, D. Antón-García, M. H. Frosz, E. Reisner, T. G. Euser**

SUPPLEMENTARY INFORMATION

Contents

Experimental Methods 2
DFT Calculations 2
Optimized Geometries 3
Supplementary Figures 9
Figure S1 9
Figure S2 9
Figure S3 10
Figure S4 11
Figure S5 12

Experimental Methods

All chemicals and reagents were purchased from commercial suppliers and used as received unless otherwise noted. Laboratory-grade reagents were used for synthesis, and chemicals for the analytical part were of the highest available purity. Aqueous $[Ru(bpy)_3]^{2+}$ stock solutions were made using tris(2,2-bipyridyl)dichlororuthenium(II) hexahydrate (99.95% purity, Sigma-Aldrich). Phosphate buffer solutions (1.0 M) were prepared by titration to the desired pH, as determined by a pH electrode (Mettler Toledo, SevenEasy).

Samples were purged under a nitrogen atmosphere for 15 minutes before being extracted into a gas-tight syringe and injected into the HC-PCF using a syringe pump (Aladdin AL-10). Repeat measurement runs were loaded successively into the fiber and started once the spectrum returned to the reference baseline. This ensured that residual reaction products from the previous run had been pushed clear of the fiber. The fiber was cleaned through with water between different sample sets, and subsequent samples were loaded once the spectrum matched an internal water reference. Quantitative background-subtracted absorption spectra were obtained by referencing each spectrum to that of the initial, unirradiated sample. Spectra were acquired using an Ocean Optics QE65000 spectrometer.

DFT Calculations

Fully optimized molecular structures were obtained through geometry optimizations applying the B3LYP[1–3] density functional in conjunction with the 6-31G(d,p)[4–6] basis set. Solvation was approximated by the conductor-like model CPCM[7] with the refractive index and dielectric constant set to water. Long-range Coulomb and HF exchange interactions were included using the *rijcosx* approximation. All calculations were conducted using ORCA 4.0.1.2.[8]

References

(1) Lee, C.; Yang, W.; Parr, R. G. Development of the Colle-Salvetti Correlation-Energy Formula into a Functional of the Electron Density. *Phys. Rev. B* **1988**, *37* (2), 785–789. https://doi.org/10.1103/PhysRevB.37.785.

(2) Becke, A. D. Density-Functional Exchange-Energy Approximation with Correct Asymptotic Behavior. *Phys. Rev. A* **1988**, *38* (6), 3098–3100. https://doi.org/10.1103/PhysRevA.38.3098.

(3) Becke, A. D. Density-functional Thermochemistry. III. The Role of Exact Exchange. *J. Chem. Phys.* **1993**, *98* (7), 5648–5652. https://doi.org/10.1063/1.464913.

(4) Francl, M. M.; Pietro, W. J.; Hehre, W. J.; Binkley, J. S.; Gordon, M. S.; DeFrees, D. J.; Pople, J. A. Self-consistent Molecular Orbital Methods. XXIII. A Polarization-type Basis Set for Second-row Elements. *J. Chem. Phys.* **1982**, *77* (7), 3654–3665. https://doi.org/10.1063/1.444267.

(5) Rassolov, V. A.; Pople, J. A.; Ratner, M. A.; Windus, T. L. 6-31G * Basis Set for Atoms K through Zn. *J. Chem. Phys.* **1998**, *109* (4), 1223–1229. https://doi.org/10.1063/1.476673.

(6) Hehre, W. J.; Ditchfield, R.; Pople, J. A. Self—Consistent Molecular Orbital Methods. XII. Further Extensions of Gaussian—Type Basis Sets for Use in Molecular Orbital Studies of Organic Molecules. *J. Chem. Phys.* **1972**, *56* (5), 2257–2261. https://doi.org/10.1063/1.1677527.

(7) Barone, V.; Cossi, M. Quantum Calculation of Molecular Energies and Energy Gradients in Solution by a Conductor Solvent Model. *J. Phys. Chem. A* **1998**, *102* (11), 1995–2001. https://doi.org/10.1021/jp9716997.

(8) Neese, F. The ORCA Program System. *WIREs Comput. Mol. Sci.* **2012**, *2* (1), 73–78. https://doi.org/10.1002/wcms.81.

Optimized Geometries

xyz-coordinates of optimized structures with -py referring to the addition of an axial pyridine ligand and -H referring to the hydride.

Co(I)

Co	-6.75807967681045	17.96476206334768	-17.79809835136351
N	-7.44303702757813	19.66739370222275	-17.52960400730797
C	-6.86205935912897	20.41717488579585	-16.62631169502008
C	-5.72426921095435	19.74121276097280	-16.00690997481007
N	-5.54656254108269	18.52172318565290	-16.48199842224203
O	-8.54685552286282	20.18611326014904	-18.17082419030412
N	-7.96997067173668	17.40762357524986	-19.11377741746505
C	-7.79209787925585	16.18820122129577	-19.58907955158518
C	-6.65385295819938	15.51257092438690	-18.97019044010219
N	-6.07305198365377	16.26235054795885	-18.06680886844964
O	-8.98238793185050	18.14772856555470	-19.56110773190250
O	-4.96877266486689	15.74385184889488	-17.42617454955609
O	-4.53470674652801	17.78125875689435	-16.03398027241232
H	-4.71453600699861	16.54222461625886	-16.79124073261217
H	-8.80167783749479	19.38729731467607	-18.80497493694388
C	-7.33310897185620	21.79120092013249	-16.25984360724281
H	-8.11989739792844	21.74587719141788	-15.49542917815452
H	-6.51790942393335	22.39479317987708	-15.85548130089593
H	-7.75911220606202	22.29182387916366	-17.13243883812108
C	-4.83837534152368	20.35715639677401	-14.97153152889203
H	-5.41125868792068	20.96854261345558	-14.26773282049956
H	-4.31845835589687	19.56739706534545	-14.42571125965050
H	-4.07768108221744	21.00703157600147	-15.42589225678782
C	-6.18222062194138	14.13883633948673	-19.33678249388696
H	-5.39359407983841	14.18431840328413	-20.09927995700567
H	-5.75842712722350	13.63725170378395	-18.46362897837672
H	-6.99670873424479	13.53584429779149	-19.74342755095525
C	-8.67857234148832	15.57232444320372	-20.62398463801573
H	-8.10618511716036	14.96048062773327	-21.32780269355711
H	-9.43966061947582	14.92314330645088	-20.16926981551048
H	-9.19811214528685	16.36227722278692	-21.16990878237101

Co(I)-py

Co	-6.91907681015857	17.82481484170056	-17.45849751237359
N	-7.62419681440443	19.56918401066500	-17.25345800968898
C	-6.89703519593146	20.39670480398553	-16.51831023354423
C	-5.61133617930332	19.84090728358749	-16.14114512271155
N	-5.42860908847607	18.60571276314757	-16.58738089800828
O	-8.85266775842609	19.93531290279423	-17.63679610784287
N	-8.08667729767230	17.30381861283125	-18.80599744449377
C	-7.87907273217099	16.11160675775421	-19.37935774382587
C	-6.64443564293075	15.53348792242918	-18.94886700856511
N	-6.00492559508377	16.33957104273157	-18.09413961161122
O	-9.30617577790326	17.90757784029102	-19.06266430115825
O	-4.83072680471691	15.85493423312047	-17.54781324782804
O	-4.28640803191212	17.95878305612172	-16.31144858528119
H	-4.50808415119523	16.70038814903392	-17.00219505875220
H	-9.20232813628741	18.79820460236554	-18.51791132521161
C	-8.59299151685876	15.94966332394671	-16.01551075382708
C	-9.31350931269743	15.39577152307074	-14.96366219631715
C	-9.24513028142893	15.99743853042046	-13.70602456424474
C	-8.45524589216969	17.13824515416420	-13.55600521680359
C	-7.78167563719566	17.64304866849859	-14.66280307562319
N	-7.84085759126984	17.05814905721681	-15.87382014398838
H	-8.60657388876627	15.50311377680645	-17.00552701299774
H	-9.91343412562309	14.50882040523668	-15.13688457040413

H	-9.79391160592422	15.58635052170244	-12.86485651477392
H	-8.36093352492521	17.64168892569597	-12.59978743846095
H	-7.17431036716298	18.53973240065241	-14.59621623099867
C	-6.06551503333197	14.22241295979220	-19.38885259712407
H	-5.77951951075927	13.61477183729456	-18.52365081511029
H	-6.77474433997316	13.65439603334560	-19.99274075928642
H	-5.15527114330138	14.36590399600781	-19.98497170508730
C	-8.84554494960681	15.51766945981527	-20.35688495251143
H	-8.39122934371193	14.69476987147323	-20.91209704771729
H	-9.74372724229425	15.13573880439275	-19.85479657796319
H	-9.17961198785025	16.27573352319425	-21.07256189815863
C	-4.56532954707775	20.56250296035782	-15.34758221125379
H	-5.01366304502845	21.27305580125385	-14.64848152039955
H	-3.96817601067175	19.83623808431308	-14.79089845756648
H	-3.87769298737155	21.12388139394975	-15.99535101460437
C	-7.40511747121354	21.75674095589675	-16.14196479550484
H	-7.86061139582724	22.23958091528435	-17.01284330410027
H	-8.19126248327853	21.69350683667350	-15.37819036727756
H	-6.60938835110739	22.39420915898457	-15.75169980599820

Co(II)

Co	-6.81710346759257	17.95570601892818	-17.78018044632791
N	-7.62057717303702	19.63906174453700	-17.38385199797014
C	-6.92594067020574	20.41938650786012	-16.58667304798646
C	-5.69156309442327	19.79028366043183	-16.09441796089899
N	-5.48647027321584	18.60612009521324	-16.59110767365520
O	-8.76544824801899	20.02990862991496	-17.90615613426103
N	-8.15062035783433	17.30223714948135	-18.95928543274552
C	-7.93530683859735	16.12633780372531	-19.47475300459919
C	-6.70151202610754	15.49482432321251	-18.98434859921567
N	-6.01834333530623	16.26579445362554	-18.16929120810839
O	-9.27639608856693	17.99269443466057	-19.31379524514298
O	-4.89327150988859	15.86254091944029	-17.61452209244025
O	-4.36706569253789	17.91080412760126	-16.22498895326595
H	-4.49257056627406	17.02222015447579	-16.75802966941448
O	-5.74317667991008	18.91153238429871	-19.53642649316476
H	-9.15768055946789	18.87647303107367	-18.76757705117354
H	-8.40798049114886	16.25084192636962	-16.29119517028019
H	-7.07015502370938	16.40578095608857	-15.57305021349456
O	-7.78581182465510	16.92146299174626	-15.97319755511418
H	-6.28283843075179	18.84914115140035	-20.33767442378737
H	-5.64922410811091	19.86278018131931	-19.38176877136484
C	-7.34482069348961	21.80983729696880	-16.22806921563241
H	-8.18739092267696	22.09748304401860	-16.85714192594745
H	-7.65583341308705	21.88450006307397	-15.17872092903738
H	-6.52387159338867	22.51773126189944	-16.38257983658661
C	-4.76558903821303	20.45476724466828	-15.12674436066939
H	-5.32520596145533	21.06026812784038	-14.41003476128940
H	-4.17752242523328	19.70815465308181	-14.59078317029172
H	-4.07314989605430	21.12027675932080	-15.65667785978344
C	-6.28210456500686	14.10580563482796	-19.34886974326095
H	-5.39951823331151	13.84004345276238	-18.76705232634954
H	-7.07984253772643	13.38631721044249	-19.13463542974456
H	-6.03685184090340	14.02184562682588	-20.41435295451528
C	-8.85845797735098	15.47072211894307	-20.45058187745863
H	-8.29852526380233	14.86195264087054	-21.16399448792094
H	-9.56038032395081	14.81058470200499	-19.92637689906897
H	-9.43698473698908	16.22292440504632	-20.98932994803181

Co(II)-py

Co	-6.81710346759257	17.95570601892818	-17.78018044632791

N	-7.62057717303702	19.63906174453700	-17.38385199797014
C	-6.92594067020574	20.41938650786012	-16.58667304798646
C	-5.69156309442327	19.79028366043183	-16.09441796089899
N	-5.48647027321584	18.60612009521324	-16.59110767365520
O	-8.76544824801899	20.02990862991496	-17.90615613426103
N	-8.15062035783433	17.30223714948135	-18.95928543274552
C	-7.93530683859735	16.12633780372531	-19.47475300459919
C	-6.70151202610754	15.49482432321251	-18.98434859921567
N	-6.01834333530623	16.26579445362554	-18.16929120810839
O	-9.27639608856693	17.99269443466057	-19.31379524514298
O	-4.89327150988859	15.86254091944029	-17.61452209244025
O	-4.36706569253789	17.91080412760126	-16.22498895326595
H	-4.49257056627406	17.02222015447579	-16.75802966941448
O	-5.74317667991008	18.91153238429871	-19.53642649316476
H	-9.15768055946789	18.87647303107367	-18.76757705117354
H	-8.40798049114886	16.25084192636962	-16.29119517028019
H	-7.07015502370938	16.40578095608857	-15.57305021349456
O	-7.78581182465510	16.92146299174626	-15.97319755511418
H	-6.28283843075179	18.84914115140035	-20.33767442378737
H	-5.64922410811091	19.86278018131931	-19.38176877136484
C	-7.34482069348961	21.80983729696880	-16.22806921563241
H	-8.18739092267696	22.09748304401860	-16.85714192594745
H	-7.65583341308705	21.88450006307397	-15.17872092903738
H	-6.52387159338867	22.51773126189944	-16.38257983658661
C	-4.76558903821303	20.45476724466828	-15.12674436066939
H	-5.32520596145533	21.06026812784038	-14.41003476128940
H	-4.17752242523328	19.70815465308181	-14.59078317029172
H	-4.07314989605430	21.12027675932080	-15.65667785978344
C	-6.28210456500686	14.10580563482796	-19.34886974326095
H	-5.39951823331151	13.84004345276238	-18.76705232634954
H	-7.07984253772643	13.38631721044249	-19.13463542974456
H	-6.03685184090340	14.02184562682588	-20.41435295451528
C	-8.85845797735098	15.47072211894307	-20.45058187745863
H	-8.29852526380233	14.86195264087054	-21.16399448792094
H	-9.56038032395081	14.81058470200499	-19.92637689906897
H	-9.43698473698908	16.22292440504632	-20.98932994803181

Co(III)

Co	-6.79683663741844	17.95439365817356	-17.78296999484167
N	-7.61860174137800	19.64212629244694	-17.36974346333040
C	-6.93188066875108	20.40837333121870	-16.55822129462949
C	-5.66435139974258	19.80765876620514	-16.12025826837489
N	-5.44664239550505	18.62776085151363	-16.61608391497580
O	-8.78608055387696	19.98151048441984	-17.83268631488704
N	-8.15178977784664	17.28993152187318	-18.94452968414036
C	-7.92765893069722	16.11541892644299	-19.45601600506502
C	-6.69498226649620	15.48127102347839	-18.96649737691342
N	-5.99176721532042	16.25210992955570	-18.17333102435855
O	-9.25027623485739	17.99545138234662	-19.30610054341192
O	-4.87499808035665	15.88201625084538	-17.62029542197714
O	-4.29620392457623	17.96919370056829	-16.33332960539652
H	-4.42294070148872	17.06310453747637	-16.82576029698057
O	-5.84959694185472	18.72795231409265	-19.26691407919363
H	-9.16404407740640	18.86759633470276	-18.73952878908392
H	-8.46289665636717	16.66377410255518	-16.45706136205555
H	-7.09441395847857	16.58467955505259	-15.73113434866426
O	-7.66619283537081	17.17865249536611	-16.24552965043775
H	-6.04889559518113	18.33792594144219	-20.13424395966759
H	-5.96123818867822	19.68782013440112	-19.36764968915301
C	-7.43089796054805	21.74886197828163	-16.12433039511311
H	-7.93362494524657	22.23613440630185	-16.96383163852419
H	-8.16568123895724	21.64709798387377	-15.31695181253677

H	-6.61802273527099	22.38192605044283	-15.76731683408345
C	-4.70804791851922	20.49911504635136	-15.20788470332014
H	-5.24782302940491	21.06969565701585	-14.44954519711530
H	-4.05081746311820	19.77541528919897	-14.72593635166804
H	-4.09177550959214	21.20021349643254	-15.78365445931115
C	-6.29413092547439	14.08507869126240	-19.31293762640642
H	-5.46833432908241	13.77901149712251	-18.67148986094776
H	-7.13506043776265	13.39978111442910	-19.17006498528564
H	-5.97098311134020	14.01119840922301	-20.35775921075565
C	-8.83968628924427	15.46141662740418	-20.43848392846368
H	-8.26659802647085	14.86395858406576	-21.15024989645619
H	-9.52944384401267	14.79026275221431	-19.91297919512948
H	-9.42558159530662	16.20976891420278	-20.97306153734453

Co(III)-py

Co	-6.88624259039048	17.90046335791121	-17.59729411490024
N	-7.71224064497477	19.59301779476628	-17.24937075427418
C	-6.98381978685833	20.43479015968368	-16.55683487366466
C	-5.67728715701539	19.89046651142453	-16.16295366263663
N	-5.50329362159366	18.65377224855823	-16.52008651952560
O	-8.91536143622819	19.87721678751803	-17.65641082806507
N	-8.16580392398921	17.24316084219302	-18.84931570924706
C	-7.87053759777694	16.10796797794498	-19.40992593144178
C	-6.65839668503205	15.47888218934227	-18.86265913783759
N	-6.03726266002417	16.22408108587894	-17.97843385379291
O	-9.27210382459320	17.92984508941921	-19.22247034062997
O	-4.94941805614498	15.85345667304053	-17.36966653266603
O	-4.35561640813311	18.00477872919050	-16.21030886476353
H	-4.50171930289853	17.06451656596801	-16.63387156059331
O	-5.95066443920987	18.71209926125685	-19.11368257180693
H	-9.24144917168348	18.77279106910423	-18.60906225819666
C	-8.71643595915612	16.12754382165055	-16.22732261528620
C	-9.37931834933168	15.53522208805548	-15.16169544177504
C	-9.09141067888469	15.95068826763543	-13.86338610461703
C	-8.14421925765495	16.95762938161277	-13.68917661552041
C	-7.52652919726711	17.51168105283090	-14.80168517406807
N	-7.79732815901959	17.10503575293549	-16.06189165800409
H	-8.93326847836389	15.81092389570881	-17.23396507896846
H	-10.10731618462588	14.75931175390102	-15.36730687398978
H	-9.59287741275632	15.50131258343684	-13.01309384359633
H	-7.87286173479610	17.32760606372372	-12.70720264334734
H	-6.79608476844224	18.29318124527520	-14.67657523770941
H	-5.49273615229038	18.07646435954754	-19.68772688285293
H	-6.51940132502799	19.23614654816505	-19.70095696843709
C	-7.48532095741608	21.79333943942940	-16.18749466880525
H	-8.00893062608738	22.23354217368978	-17.04046335329695
H	-8.20509679199626	21.72574013259688	-15.36311122309108
H	-6.67289210200719	22.45069286299001	-15.87774526114571
C	-4.67054334384978	20.67465732519651	-15.39034081149604
H	-5.15857153064761	21.23121887514160	-14.58632402284269
H	-3.91367003083062	20.01352305295329	-14.97008587833307
H	-4.17989921367371	21.40031513520281	-16.04944539793637
C	-6.19870674211688	14.10863680624230	-19.24014840230570
H	-5.33400108033849	13.84027263911434	-18.63448783857445
H	-6.99602697770626	13.37818675922683	-19.06814419711873
H	-5.91911426192981	14.05834233223779	-20.29804196368738
C	-8.71657443398588	15.46368098009237	-20.45515174882311
H	-8.10014202714667	14.89333221206782	-21.15220629568702
H	-9.41866571551629	14.76731470470897	-19.97970588999756
H	-9.29227419358778	16.21327160042990	-20.99883493164477

Co(II)-H

Co	-6.89749370706332	17.85907988886736	-17.61142170551609
N	-7.52628321343627	19.62966288274892	-17.46708007591186
C	-6.84524936108862	20.43156714098128	-16.68639384925142
C	-5.62853500506398	19.81753961083686	-16.17833320554564
N	-5.48520692684297	18.55982074090779	-16.56247563118253
O	-8.71560378334138	20.08267962436803	-17.97957212050489
N	-8.08417325815560	17.32776035759354	-18.98182088396168
C	-7.79183075483405	16.18729287000380	-19.58372437090125
C	-6.58922884576600	15.55899792604471	-19.05630113232493
N	-6.01903129906299	16.27166147924674	-18.11524796736347
O	-9.16487392037086	18.00973935434809	-19.32948216222584
O	-4.91449624637245	15.75444319695964	-17.48202441655780
O	-4.41379813971895	17.86602300605330	-16.20993186368620
H	-4.61115817093045	16.57949159321595	-16.91324587484849
H	-8.99211727349901	19.27603818888866	-18.58824486300325
H	-7.73995124868471	17.24400543033245	-16.41903438994936
C	-7.31383143383731	21.80842400563016	-16.32678942019754
H	-7.79158836378731	22.28394435903704	-17.18688866702646
H	-8.06410661136799	21.76033379331855	-15.52691541548627
H	-6.49123318682316	22.43246813534083	-15.97461874217431
C	-4.62837817465643	20.53022729311980	-15.32292699741218
H	-5.12143442343141	21.12053002063960	-14.54433593031904
H	-3.96796378884148	19.79786823404740	-14.85608281859745
H	-4.01064883576681	21.21869336985002	-15.91449235660218
C	-6.07897832456984	14.22021470129795	-19.49058147405110
H	-4.99686735237164	14.16698971621477	-19.35604425209688
H	-6.53098182191356	13.42310311173179	-18.88723214131920
H	-6.32257959228565	14.02270725116182	-20.53661507106727
C	-8.65767298662727	15.60817444564192	-20.65744924548319
H	-8.07515718922459	15.03257326323369	-21.38086078653094
H	-9.41862088268135	14.93832558100111	-20.23498190684586
H	-9.18088621258258	16.41491985533641	-21.17563441205540

Co(III)-H

Co	-6.84016053899847	17.91040098282709	-17.70350533492630
N	-7.52265293785018	19.64638659853445	-17.44563020224236
C	-6.87866221781102	20.39683384337666	-16.60014124681448
C	-5.73062847892639	19.71986685206577	-15.98276426966456
N	-5.58100468038046	18.48114621031908	-16.39192989952548
O	-8.60889205499442	20.15919389164878	-18.08626408854590
N	-8.08281797032495	17.35438242430296	-19.03323179538857
C	-7.85621496138384	16.16634564331491	-19.54290482682637
C	-6.68130576229335	15.50144431857650	-18.96181425182438
N	-6.10550775626700	16.20351137030146	-18.02948318535854
O	-9.09979506628083	18.08866419357156	-19.41491580390932
O	-4.99675903429185	15.71005132292554	-17.41275938101638
O	-4.60684221667278	17.71762036141652	-15.95915749656452
H	-4.75324929569247	16.48114999447207	-16.74914621212371
H	-8.91027361327826	19.35419761579840	-18.68395082917839
H	-7.69123943034285	17.38839020319047	-16.68044771967016
C	-8.72710423023697	15.54160455354923	-20.58132966309843
H	-8.13161779992272	15.00226714908585	-21.32273948006742
H	-9.41834459050943	14.82270670817499	-20.12277311207612
H	-9.31676338799539	16.31391056998939	-21.07692161028163
C	-6.18799508758180	14.16677627498921	-19.41636373261015
H	-5.52564385455092	14.29023557810676	-20.28209458354474
H	-5.61853927951320	13.68150470181266	-18.62320762067162
H	-7.01690903706191	13.52718999228052	-19.72503830513997
C	-7.27525588808505	21.80471264211181	-16.30071226865795
H	-8.34180969651931	21.85111441431103	-16.05941657481501

H	-6.70251071580530	22.21211918221916	-15.46918266672437
H	-7.10966051005635	22.43446499724832	-17.18230044282873
C	-4.81636648103562	20.38601509444732	-15.00911083291165
H	-5.37869836455057	20.83330583326934	-14.18300747841921
H	-4.11303529668340	19.65474509093277	-14.61145144516732
H	-4.25317748710297	21.18977228782937	-15.49866976940648

Co(II)-py-H

Co	-6.87894188136896	17.95298428599457	-17.64691193106649
N	-7.71803395855522	19.62313321479180	-17.32490940299267
C	-7.03624046728303	20.43539775634753	-16.54620956895599
C	-5.72979875632308	19.91268784235184	-16.14466422919882
N	-5.47899981803393	18.70486318563999	-16.59850917949644
O	-8.92541111142216	19.93669467703523	-17.76063420800307
N	-8.18095123026920	17.22533997732725	-18.82911342640350
C	-7.82824669889186	16.06396081577270	-19.42740113097453
C	-6.70760353565754	15.45316589912696	-18.81070260910335
N	-6.02039314564124	16.30203153669640	-18.03046055073960
O	-9.05073399884769	18.05111999131939	-19.55335404690834
O	-5.08310845510778	15.75504838723542	-17.15842463905281
O	-4.34721012713189	18.07816877992378	-16.32748070156939
H	-4.68795732539032	16.61103179413179	-16.76735495591415
H	-6.20915972799335	18.58291951803174	-18.81393441169318
H	-9.13773491777294	18.82874443012810	-18.90421613724044
C	-8.66017676239094	16.12181807007280	-16.11093600067257
C	-9.26227549585449	15.48812729047299	-15.02683332725620
C	-8.92648827131715	15.89086097101040	-13.73451240790066
C	-7.99357068003717	16.91708632807007	-13.58333745961816
C	-7.44080207465851	17.49850130314103	-14.72039966438112
N	-7.76346878164825	17.11635285790802	-15.97006028127868
H	-8.89562534305714	15.84234009232184	-17.12952503106140
H	-9.98089166145910	14.69531884190251	-15.20451023742750
H	-9.38013029622562	15.41657058914449	-12.86972720338667
H	-7.69053351063590	17.27386353363675	-12.60442986035827
H	-6.71779010504611	18.29799832225271	-14.63310257102856
C	-6.34463692498707	14.00236701909180	-18.90880063222052
H	-6.55001602498644	13.48867957148335	-17.95983173844554
H	-6.92204535704138	13.49852106227854	-19.68513520576090
H	-5.27864735369688	13.86690465737698	-19.12420104458690
C	-8.58379753705232	15.52303153325693	-20.60566895032504
H	-8.10360655653830	14.62882436035549	-21.00657098122533
H	-9.62121128717429	15.26792793993587	-20.34987259866693
H	-8.62894044714945	16.26805095785921	-21.40794746718175
C	-7.57861328723553	21.76357026728640	-16.11397853908763
H	-8.25203205864706	22.14490958510951	-16.88521873148928
H	-8.16127496884546	21.68156605193314	-15.18674304801695
H	-6.77846363442693	22.48551494940108	-15.93514809591964
C	-4.78331804597027	20.64498621947213	-15.24382686809727
H	-5.32667821701942	21.21937935622900	-14.48794068860789
H	-4.12854612793568	19.92266247347589	-14.75154546868748
H	-4.14890141627295	21.34760328266657	-15.79995945499846

Supplementary Figures

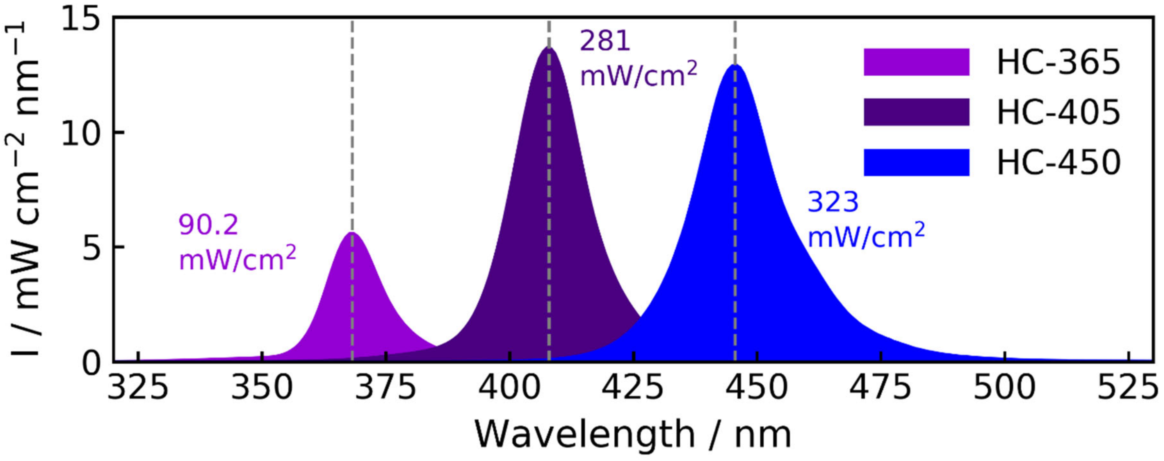

Figure S1. Spectroscopic profiles of the three excitation sources used in experiments, normalized to the incident power at the position of the fiber. Full-width half-maximum (FWHM) values of each source are 13 nm, 17 nm and 20 nm for the 365 nm, 405 nm and 450 nm sources respectively.

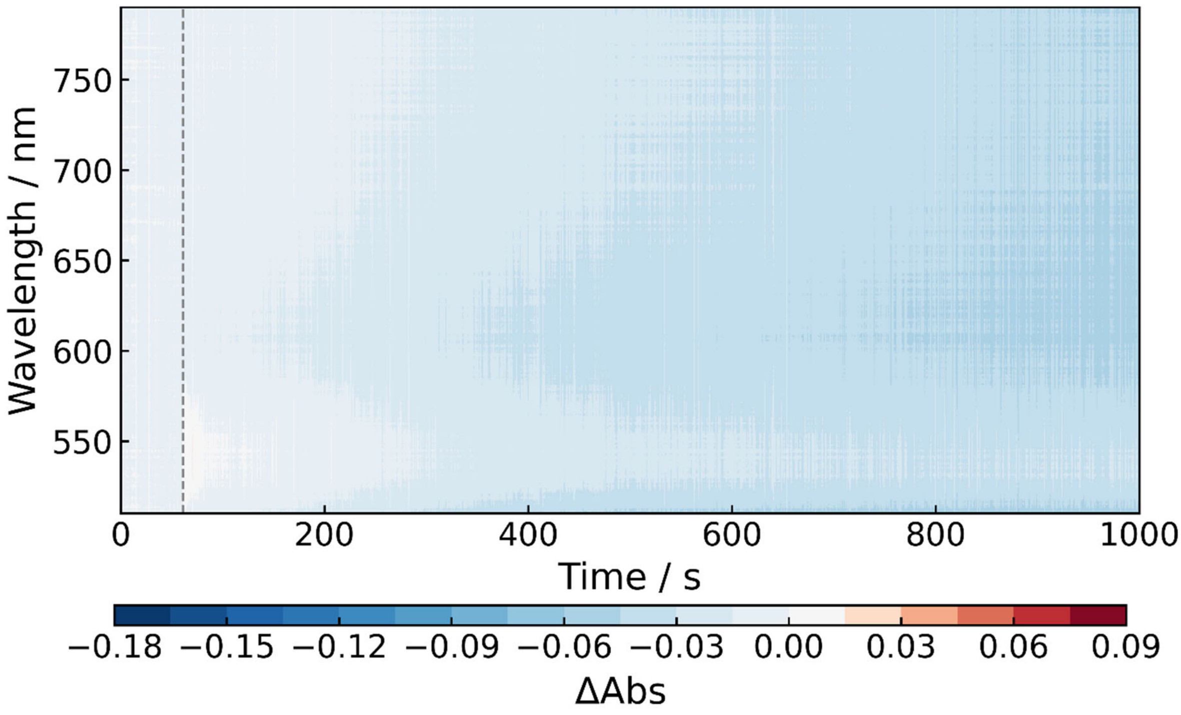

Figure S2. Cuvette-based color maps detailing the time evolution of absorption as a function of wavelength for the $[CoCl(dmgH)_2py]$ catalyst with $[Ru(bpy)_3]^{2+}$ as a photosensitizer and sodium ascorbate as an electron donor. Each sample was made up of 44 µM $[Ru(bpy)_3]^{2+}$, 86 µM $[CoCl_2(dmgH)(dmgH_2)]$, 86 µM pyridine, 0.1 M sodium ascorbate in a pH 8 phosphate buffer (0.2 M). The reaction was driven with a 450 nm LED with an irradiance of 187 mW cm^{-2}. The grey dashed line indicates the time at which the LED source was switched on.

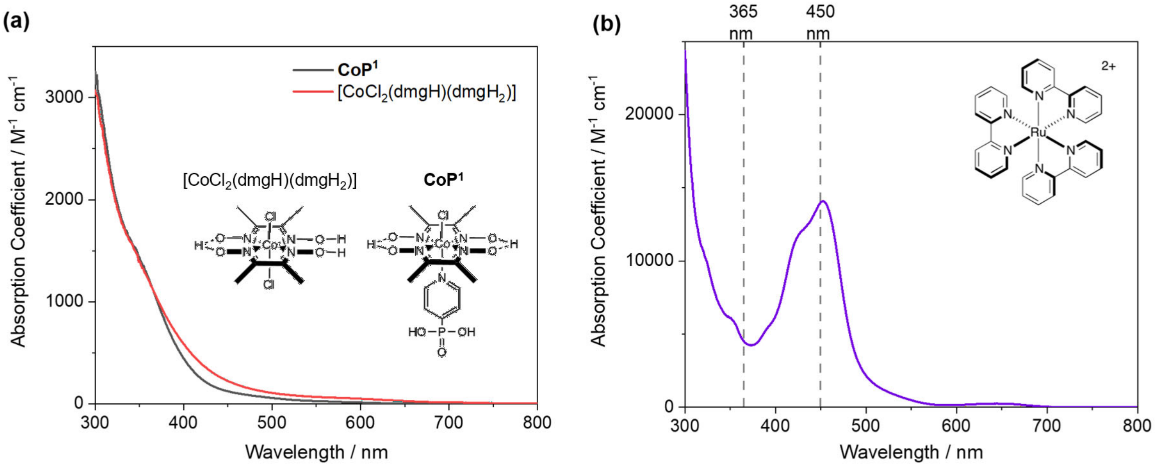

Figure S3. UV-Vis absorption spectra of; (a) cobaloximes, and (b) $[Ru(bpy)_3]^{2+}$. Measurements were performed in cuvettes and all samples were recorded in an aqueous solution. The wavelengths of the closest excitation sources to the absorption maximum are represented by the dashed lines.

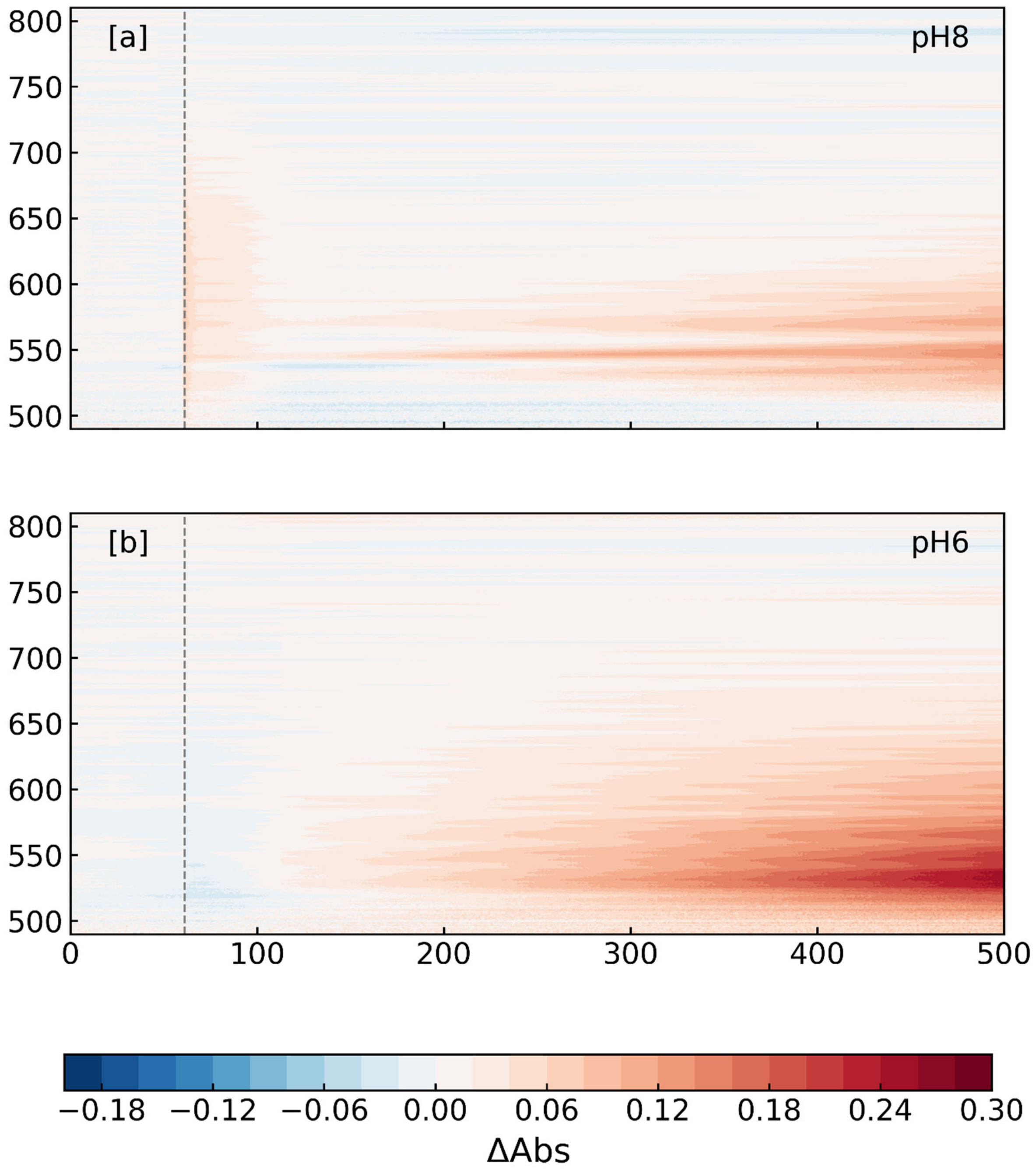

Figure S4. *In-fibra* color maps detailing the time evolution of absorption as a function of wavelength for the [CoCl(dmgH)$_2$py] catalyst with [Ru(bpy)$_3$]$^{2+}$ as a photosensitizer and sodium ascorbate as an electron donor. Each sample was made up of 44 μM [Ru(bpy)$_3$]$^{2+}$, 86 μM [CoCl$_2$(dmgH)(dmgH$_2$)], 86 μM pyridine and 0.1 M sodium ascorbate. The reaction was driven with a 450 nm LED with an irradiance of 323 mW cm^{-2}. Experiments were undertaken using a 0.2 M phosphate buffer to hold the solution at (a) pH 8 and (b) pH 6. The grey dashed line indicates the time at which the LED source was switched on.

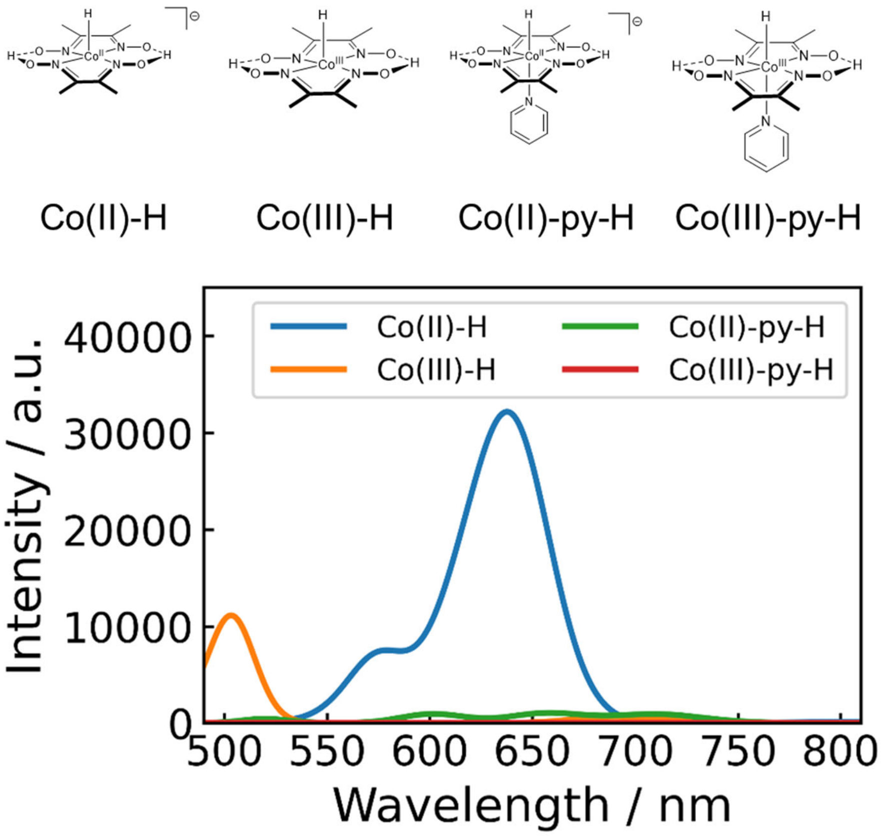

Figure S5. DFT-simulated spectra of cobaloxime hydride species using the B3LYP functional and 6-31G(d,p) basis set combination. DFT simulations were performed as described above.
